# Supplementary material for: The interactome of intact mitochondria by cross-linking mass spectrometry provides evidence for coexisting respiratory supercomplexes
Source: Mol Cell Proteomics. 2017 Dec 8;17(2):216–32. doi: 10.1074/mcp.RA117.000470 (PMC5795388; doi:10.1074/mcp.RA117.000470)
Supplement: Supplemental Data [file supp_RA117.000470_133922_0_supp_23978_fzffwf.zip › spectra_annotation/mito_DR_spectra_annotation/3-1-30-1-18-1.pdf]

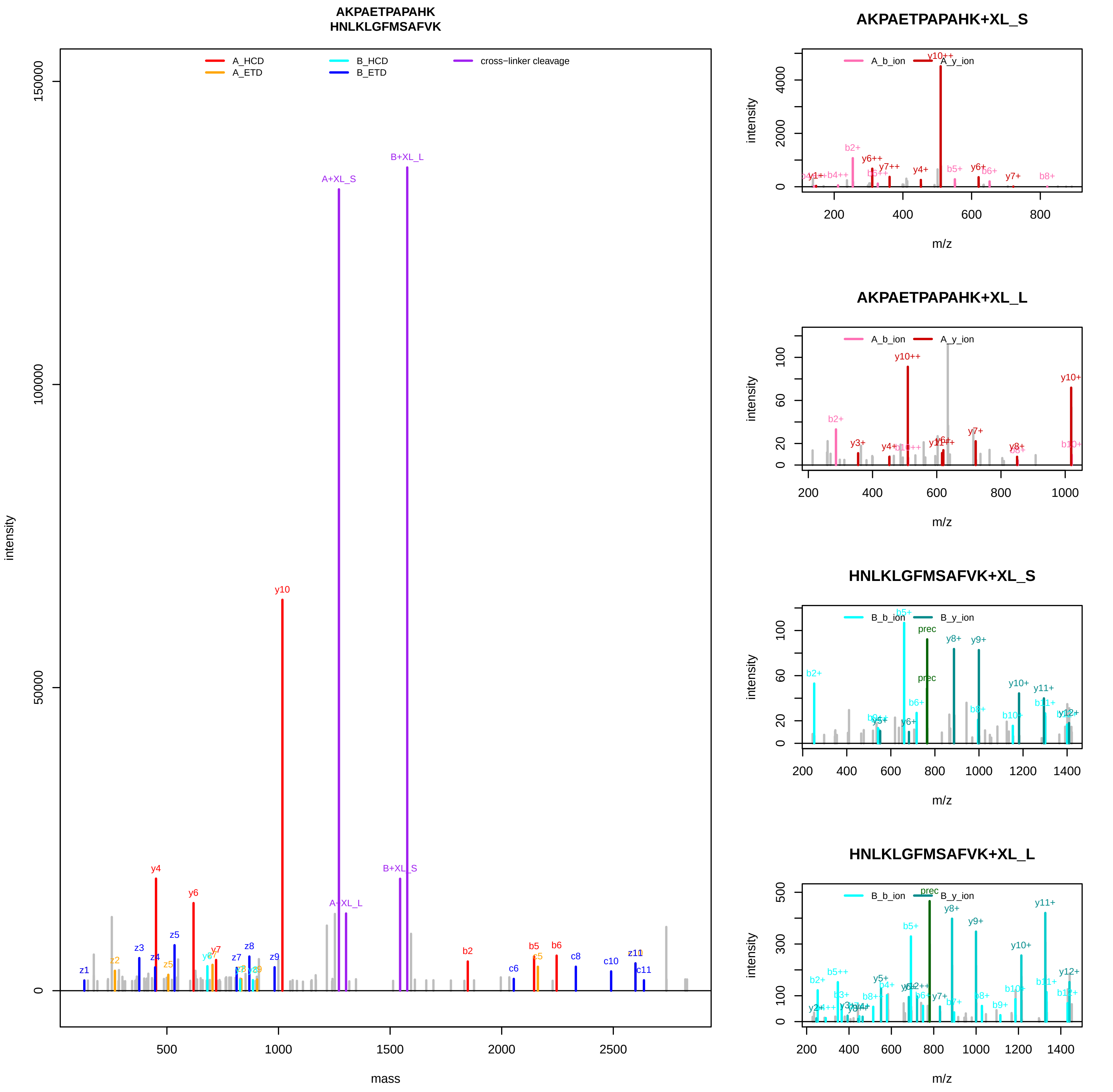
AKPAETPAPAHK
HNLKLGFMSAFVK
A_HCD
A_ETD
B_HCD
B_ETD
cross−linker cleavage
intensity
mass
150000
100000
50000
0
500
1000
1500
2000
2500
A+XL_S
B+XL_L
B+XL_S
A+XL_L
y10
y4
y6
y7
b2
b5
b6
c5
c6
c8
c10
c11
z11
z1
z2
z3
z4
z5
z7
z8
z9
AKPAETPAPAHK+XL_S
AKPAETPAPAHK+XL_L
HNLKLGFMSAFVK+XL_S
HNLKLGFMSAFVK+XL_L
A_b_ion
A_y_ion
B_b_ion
B_y_ion
prec
m/z
intensity
y10++
y10+
y9+
y8+
y11+
y12+
b5+
b2+
